# Ethical Imperatives for Retrieval-Augmented Generation in Clinical Nursing: Viewpoint on Responsible AI Use

**DOI:** 10.2196/79922

**Published:** 2026-01-09

**Authors:** Xinyi Tu, Chenghao Shi, Peilin Qian, Lizhu Wang

**Affiliations:** 1 Department of Nursing, The Second Affiliated Hospital of Zhejiang University School of Medicine, No. 88 Jiefang Road, Shangcheng District, Hangzhou, Zhejiang, 310009, China, 86 13867466291, 86 0571-87787013; 2 School of Medicine, Zhejiang University, Hangzhou, Zhejiang, China; 3 School of Nursing, Chinese Academy of Medical Sciences & Peking Union Medical College, Beijing, Beijing, China

**Keywords:** large language models, clinical decision‑making, ethics, fairness, transparency

## Abstract

Retrieval-augmented generation (RAG) systems have emerged as a powerful technique to enhance the capabilities of large language models by enabling them to access external, up-to-date knowledge in real time, and RAG systems are being increasingly adopted by researchers in the medical field. In this viewpoint article, we explore the ethical imperatives for implementing RAG systems in clinical nursing environments, with particular attention to how these technologies affect patient care quality and safety. The purpose of this paper is to examine the ethical risks introduced by RAG-enhanced large language models in clinical nursing and to propose strategic guidelines for their responsible implementation. Key considerations include ensuring accuracy, fairness, transparency, and accountability, as well as maintaining essential human oversight, as discussed through a structured analysis. We argue that robust data governance, explainable artificial intelligence (AI) techniques, and continuous monitoring are critical components of a responsible RAG implementation strategy. Ultimately, realizing the benefits of RAG while mitigating ethical concerns requires sustained collaboration among health care professionals, AI developers, and policymakers, fostering a future where AI supports patient safety, reduces disparities, and improves the quality of nursing care.

## Introduction

Advances in artificial intelligence (AI) are rapidly transforming the health care field, with large language models (LLMs) playing a pivotal role in revolutionizing nursing practice [1,2]. These models enhance human capabilities by learning patterns from large amounts of text data and generating contextually relevant information, offering benefits such as improved decision support, streamlined care planning, and personalized patient education [3]. LLMs can assist nurses by providing comprehensive insights and generating evidence-based recommendations. However, their integration into clinical workflows faces significant challenges [4], partly because LLMs do not operate like traditional rule-based programs. Instead, they generate responses based on statistical patterns learned from large text corpora, rather than performing logical reasoning or interpreting meaning as humans do.

While this generative capability allows LLMs to produce contextually relevant and fluent text, it also gives rise to several critical limitations in specialized clinical applications. These include biases inherited from training data and the “black-box” nature of their decision-making processes, which make it difficult to understand how outputs are produced. Such issues lead to concerns about trust and transparency, ultimately undermining the reliability of model-generated recommendations in high-stakes health care environments [5,6].

To address these shortcomings, the retrieval-augmented generation (RAG) system has emerged as an enhancement to LLMs. By combining external knowledge retrieval with the language patterns an LLM has learned during training, RAG can provide more accurate, up-to-date, and context-sensitive information [7]. In this paper, the term “RAG system” refers to the practical implementation of the RAG approach, in which an LLM retrieves external information from databases or documents and integrates it with the patterns learned during training to generate context-aware responses. While this approach aims to overcome the static nature of traditional LLMs, it introduces new ethical risks, particularly related to the quality and reliability of external data sources, the complexity of information traceability, and privacy concerns [4].

This article examines the ethical challenges introduced by RAG-optimized LLMs in clinical nursing and proposes strategies for responsible implementation. We focus on three core ethical imperatives: ensuring accuracy, fairness, and bias mitigation; promoting transparency, explainability, and trust; and maintaining responsibility, accountability, and oversight. By addressing these challenges proactively, we can harness the benefits of RAG to enhance nursing practice while safeguarding patient well-being.

## The Promise and Peril of RAG in Nursing

### Overview

RAG offers significant opportunities to enhance clinical nursing practice by providing more accurate, up-to-date, and context-specific information. By combining the language-pattern learning capability of LLMs with external knowledge retrieval, RAG can address some of the limitations of traditional LLMs [8]. However, alongside its potential benefits, RAG also introduces unique risks that need to be carefully considered, particularly in the context of clinical nursing.

### The Promise of RAG in Nursing

The primary advantage of RAG lies in its ability to enhance decision support by integrating real-time, relevant knowledge from external sources. In high-pressure environments, such as hospitals, where quick decision-making is critical, RAG can significantly improve the accuracy of clinical recommendations by ensuring they are based on the latest research and guidelines [7,9]. By enabling real-time access to updated information, RAG helps reduce errors that may arise from outdated or incomplete data [9].

Additionally, RAG systems build on what the LLM has learned from language data to provide personalized and context-specific recommendations by incorporating patient-specific data, such as medical history, treatment plans, and current health status. This ability to tailor recommendations to the individual needs of patients ensures that health care providers can deliver more accurate and effective care [10]. In settings with limited resources, RAG can provide vital support for nurses by offering evidence-based recommendations that might otherwise be difficult to access due to time constraints or a lack of available resources [9].

### The Peril of RAG in Nursing

#### Overview of Risks

Despite its advantages, the use of RAG systems in nursing also introduces a range of ethical challenges and risks. Many of these challenges stem from the limitations of the underlying LLMs on which RAG is built—including issues such as bias, opacity, and a lack of accountability. At the same time, RAG introduces additional, system-specific risks because it retrieves and integrates external information dynamically at query time. These additional risks center on data quality, source reliability, semantic coherence, and information provenance and must be carefully addressed in clinical applications.

#### Common Risks of LLMs

Many of the risks associated with RAG are rooted in the inherent challenges of LLMs, which include the following:

- Bias and fairness: LLMs may perpetuate biases that are present in their training data, such as racial, gender, or cultural biases [11]. Since RAG systems rely on external sources, they can also inherit and even amplify these biases if the external data sources are not sufficiently diverse or representative [4]. This is particularly concerning in nursing, where such biases can lead to unequal treatment and care disparities, potentially exacerbating health inequalities [12].
- Transparency and explainability: The “black-box” nature of LLMs remains a significant issue, making it difficult for clinicians to understand how the model produces its responses [13,14]. While RAG can improve transparency by citing the sources of external information, the process may still be opaque. This lack of explainability can undermine trust in the system, especially when nurses are unable to interpret the reasoning behind a recommendation [5,15].
- Accountability: Another concern is who should bear responsibility when a RAG system provides a recommendation that leads to an error or harm. It may be unclear who is responsible—whether it is the developers of the model, the clinical staff who implemented it, or the health care institution as a whole. This ambiguity complicates accountability and the governance of AI-driven health care systems [16].

#### Risks Specific to RAG

While many of the risks in RAG are shared with LLMs, there are several risks that are unique to RAG systems due to their reliance on outside information sources.

- Information provenance and quality: RAG systems pull data from many different websites, databases, and documents. Some of these sources may not undergo rigorous peer review, and there may be discrepancies in the information retrieved [17,18]. When the information used by RAG is incomplete or unreliable, the guidance it gives can also be misleading. It can also be difficult to check exactly where the information came from, which makes it harder for nurses to judge whether a recommendation can be trusted [19].
- Semantic integration and coherence: When a RAG system combines information drawn from many sources, the final advice may sound uneven or use different styles of wording. For a nurse using it, this means different parts of the output may suggest slightly different approaches, which can create confusion or reduce confidence in applying the recommendation [20]. Merging text generated from different materials may result in conflicting or incompatible pieces of information, which can cause confusion or misinterpretation [21]. This issue is particularly problematic in clinical contexts, where accuracy and consistency are essential for patient safety [22].
- Privacy and security risks: To offer personalized advice, RAG systems may need access to sensitive patient data, such as medical histories, to provide personalized recommendations [18,23]. If the system’s security is weak, or if it does not meet privacy standards like HIPAA (Health Insurance Portability and Accountability Act) or GDPR (General Data Protection Regulation), confidential information could be exposed. Such data breaches could harm both patients and the health care institution [5].

- Shifting trust foundations: Traditional LLMs depend only on the language relationships learned during training, but RAG depends on both internal and external sources. This means that the reliability of RAG depends not only on the model itself but also on the trustworthiness of the outside information it uses [24,25]. If that outside data is wrong or biased, the recommendations produced by the RAG system may be wrong too, reducing nurses’ confidence in using the system [18,19,26].

An example is as follows: A nurse used a RAG-based tool to check evidence for pressure injury prevention. The system retrieved the 2014 second edition of the international pressure ulcer guideline, which recommended turning the patient every 2 hours on standard foam surfaces. However, the upcoming 2025 fourth edition emphasizes individualized repositioning schedules, the use of alternating-pressure mattresses, and closer monitoring for deep tissue injury in high-risk or obese patients. Because the RAG system relied on outdated data, the nurse followed the older 2-hour rule and missed early signs of a deep tissue injury. This case shows how outdated retrieval sources can lead to substandard care and highlights the need for RAG systems to verify and update their evidence sources regularly.

## Focused Ethical Challenges

### Overview

The introduction of RAG systems into clinical nursing presents several ethical challenges that need to be carefully considered. These challenges are not only rooted in the inherent limitations of how LLMs learn from data but also amplified by the integration of external knowledge sources [18,27]. In this section, we focus on three core ethical challenges: accuracy, bias, and fairness; transparency, explainability, and trust; and responsibility, accountability, and human oversight. These challenges must be addressed to ensure that RAG-enhanced LLMs support ethically evidence-based decisions in nursing practice [28].

### Accuracy, Bias, and Fairness

One key ethical concern when using RAG systems is maintaining the accuracy of the information they generate, especially for clinical decisions [3,19]. The accuracy of RAG depends directly on how reliable and up-to-date the outside sources are [9]. If the outside sources are outdated, incomplete, or inaccurate, the systems’ recommendations may mislead nurses and lead to unsafe decisions that could harm patients [18,19]. Therefore, RAG systems should only use checked and high-quality data that fits the medical context [18].

Additionally, bias remains a significant challenge. RAG systems can carry bias from both the training data used to develop the LLMs and the outside sources it pulls information from [26,29]. These biases can manifest in various forms, such as racial, gender, or socioeconomic biases, and can show up as racial, gender, or economic differences and may cause unfair care for some patients [30,31]. In clinical settings, where health gaps already exist, AI-related bias can make these gaps worse. For example, if RAG systems draw information from databases that underrepresent minority populations, the resulting recommendations may be less accurate or effective for these groups [32].

To reduce these risks, we need continued work to make AI systems fair [16,18]. This means checking that outside sources cover different patient groups and reviewing the system’s outputs regularly for bias. Furthermore, health care institutions and developers should also focus on including a wide range of patients in AI training data and retrieval sources to avoid making health gaps worse [33,34].

### Transparency, Explainability, and Trust

Another significant ethical challenge is transparency and explainability. While RAG systems can increase transparency by showing the sources of information, the process by which these models generate their responses is still not very clear [18,35]. Because their internal workings are complex and often hidden, it can be hard for clinicians to see how external data and built-in knowledge come together to form a recommendation [36]. This lack of clarity can reduce trust in the system, since clinicians might hesitate to use a tool whose reasoning process cannot be easily interpreted [15,37,38].

Explainability is particularly important in clinical settings, where nurses and health care professionals need to understand the basis for AI-generated suggestions in order to trust them and incorporate them into their decision-making process [15,39,40]. It is essential for RAG systems to explain their answers in a way that is easy for health care professionals to understand. For example, providing confidence scores or source citations can help clinicians evaluate the reliability of the information and determine whether it fits the patient’s situation [38,41].

Enhancing transparency and explainability will not only help build trust in AI systems but also foster responsible and ethical AI adoption in clinical practice. As RAG systems become more integrated into health care systems, transparency will be key to ensuring that their use aligns with professional standards and ethical guidelines [6].

### Responsibility, Accountability, and Human Oversight

Responsibility and accountability are key ethical concerns in the context of AI-driven decision-making. One of the main challenges in using RAG systems is that responsibility can become blurred when many parties—such as AI developers, health care providers, and institutions—take part in the same process [42]. If an error occurs because of a recommendation from a RAG system, it may be unclear who should bear responsibility for it. This uncertainty can make legal and ethical management of such situations more difficult [43,44].

To address this issue, it is important to have clear guidelines that define the roles and duties of everyone involved in the AI decision-making process [45]. Health care institutions must ensure that there are well-defined accountability structures in place to determine who is responsible when AI systems make errors [6]. Furthermore, RAG systems should be designed with human supervision in mind. While they can enhance decision support, they should never replace human judgment. Nurses and clinicians must be able to step in or question the AI-generated recommendations if they believe the advice is not in the patients’ best interest [46,47].

Keeping humans actively in each stage of the process, which is often called “human-in-the-loop” oversight, is crucial to maintaining ethical standards in AI-assisted clinical care [48,49]. This approach ensures that clinical decisions are ultimately guided by human expertise, with AI acting only as a support tool. To support this approach, ongoing training for health care professionals on the use of AI systems, along with mechanisms for reporting and addressing errors, will help uphold ethical standards and ensure that the use of RAG systems benefits patients while minimizing risks.

Addressing these ethical concerns requires not only awareness but also systematic strategies to guide responsible use of RAG systems in nursing practice.

## Strategies for Responsible RAG Implementation

### Overview

Building on the ethical challenges discussed in the *Focused Ethical Challenges* section, it is essential to implement strategies that ensure the responsible and effective use of RAG systems in clinical nursing. While RAG systems offer significant advantages, such as real-time decision support and personalized care recommendations, they also introduce unique risks. To maximize the benefits of RAG and minimize its ethical concerns, we propose several key strategies for responsible implementation. These strategies are designed as direct responses to the ethical challenges identified in the *Focused Ethical Challenges* section, translating theoretical imperatives into actionable guidance for nursing practice.

### Data Governance and Bias Mitigation

Building on the discussion of bias and fairness in *Accuracy, Bias, and Fairness*, effective data governance provides the foundation for responsible RAG implementation. Because RAG systems rely on external knowledge sources, ensuring the integrity and representativeness of these data is essential. Biases in the external data used for retrieval can have a significant impact on the outputs generated by the system, potentially leading to unfair or discriminatory outcomes [12,50,51].

To mitigate these risks, bias detection and correction mechanisms must be implemented at multiple stages of the RAG system’s development and deployment. Specifically, RAG systems should incorporate bias auditing practices to identify potential biases in the external datasets used for retrieval. This includes regular audits of the data sources to ensure they are inclusive and reflect a broad range of patient demographics and clinical scenarios [12,50]. Additionally, techniques such as fairness-aware machine learning and adversarial debiasing can be employed to reduce the impact of bias in the model’s generated recommendations. By continuously monitoring and correcting for bias, health care organizations can help ensure that RAG systems provide fair and equitable information support for all patients [43].

### Explainable AI and Transparent Decision-Making

Building on the transparency challenges identified earlier, explainable AI techniques should be integrated into RAG systems to enhance transparency and help nurses and clinicians trust the information these systems produce. By improving the explainability of RAG systems, health care professionals can better assess the basis of AI-generated suggestions and integrate them into their decision-making processes [5].

One effective way to enhance transparency is by incorporating confidence scores and contextual explanations. Confidence scores provide an indication of how certain the system is about a given output, giving clinicians a clearer understanding of the model’s reliability [52]. Contextual explanations, on the other hand, help clarify the basis for a given response by linking it to relevant patient data or clinical guidelines. For instance, a RAG system might explain that a particular medication recommendation is based on the patient’s medical history, current health status, and recent clinical studies [18]. This additional layer of information will not only improve trust in the system but also empower nurses to make informed decisions based on AI recommendations [47].

### Operationalizing Human Oversight and Collaborative Intelligence

Extending the discussion of accountability from the *Responsibility, Accountability, and Human Oversight* section, it is crucial to emphasize the role of human-in-the-loop oversight. RAG systems should not replace human judgment but rather augment it. Nurses, doctors, and other health care professionals should remain central to the decision-making process, with RAG systems serving as assistive tools to support clinical judgment rather than a substitute for clinical expertise [9,19,26].

To ensure the effectiveness of human-AI collaboration, health care organizations should establish clear guidelines for collaborative intelligence. This includes defining roles and responsibilities for both human and AI components of the decision-making process. Nurses and clinicians should be trained to understand the strengths and limitations of RAG systems and to intervene when they believe the AI-generated recommendation is not appropriate for the patient’s context [6,37,53]. In addition, RAG systems should be designed to provide options for clinicians to modify or override system-generated outputs when necessary, allowing for greater flexibility and ensuring that human expertise is always part of the decision-making process [19,54,55].

### Continuous Monitoring and Evaluation

To ensure the ongoing effectiveness and ethical integrity of RAG systems, continuous monitoring and evaluation are essential. After RAG systems are deployed in clinical practice, they must be regularly assessed for both technical accuracy and ethical impact. This process should include performance audits, where the system’s outputs and their clinical relevance are evaluated against real-world data. Continuous monitoring should also include version control and scheduled updates of external data sources to prevent outdated or inconsistent information from influencing clinical recommendations [56].

Moreover, it is equally important to assess the ethical implications of RAG systems over time. This involves gathering feedback from health care professionals who use the system, as well as patients who are impacted by the decisions. Implementing a structured feedback mechanism is critical to identify potential ethical risks such as new biases, privacy concerns, or trust issues that may arise after the model has been in use for some time [6,57]. By continuously monitoring the performance and ethical consequences of RAG systems, health care organizations can make necessary adjustments to ensure that these systems remain aligned with ethical standards and improve patient care without causing harm.

## Conclusion

RAG systems have the potential to significantly enhance nursing practice by providing accurate, context-aware, and timely information support for clinical decisions. By integrating external knowledge sources into clinical reasoning, RAG can improve patient outcomes, particularly in high-pressure environments where rapid, evidence-based decisions are crucial. However, as with any emerging technology, the deployment of RAG systems in clinical settings must be approached with caution, taking into account both the opportunities and the ethical risks they introduce.

The ethical challenges associated with RAG, including issues of accuracy, fairness, transparency, explainability, accountability, and human oversight, must be proactively addressed to ensure that these systems are used responsibly. By implementing robust data governance, ensuring the explainability of AI recommendations, maintaining human-in-the-loop oversight, and continuously monitoring the model’s performance and ethical impact, health care organizations can harness the benefits of RAG while minimizing the potential risks.

Ultimately, responsible RAG implementation will require collaboration between health care professionals, AI developers, and policymakers. By prioritizing ethical considerations in the deployment of RAG systems, we can ensure that they support patient safety, reduce health care disparities, and contribute to the overall improvement of nursing practice. Through careful, responsible integration, RAG systems have the potential to transform the future of clinical decision-making and enhance the quality of care delivered to patients worldwide.

## Abbreviations

AI: artificial intelligence
GDPR: General Data Protection Regulation
HIPAA: Health Insurance Portability and Accountability Act
LLM: large language model
RAG: retrieval-augmented generation
